# Dynamic Assessment of Hematological Parameters as Predictive Biomarkers for Disease Severity and Prognosis in COVID-19 Patients: A Longitudinal Study

**DOI:** 10.7759/cureus.63593

**Published:** 2024-07-01

**Authors:** Aparna P Patange, Jabbar V Desai, Bhupal Pujari, Aparna Marwah, Animesh Dey

**Affiliations:** 1 Department of Medicine, Krishna Institute of Medical Sciences, Krishna Vishwa Vidyapeeth (Deemed to be University), Karad, IND; 2 Department of Management Studies, Bharati Vidyapeeth (Deemed to be University) Institute of Management and Research, New Delhi, IND; 3 Department of Allied Health Sciences, Brainware University, Kolkota, IND

**Keywords:** coagulation profiles, inflammatory markers, prognosis, disease severity, hematological parameters, sars-cov-2, covid-19

## Abstract

Background: The coronavirus disease 2019 (COVID-19) pandemic, caused by severe acute respiratory syndrome coronavirus 2 (SARS-CoV-2), has led to substantial morbidity and mortality worldwide. Hematological abnormalities are common in COVID-19 patients and play a significant role in disease pathogenesis and prognosis.

Objective: This study aimed to longitudinally monitor hematological parameters in COVID-19 patients and investigate their predictive value for disease severity and prognosis.

Methods: A prospective longitudinal design was employed to enroll 121 adult patients diagnosed with COVID-19 based on positive SARS-CoV-2 reverse transcription-polymerase chain reaction (RT-PCR) test results. Baseline demographic and clinical data were collected, and hematological parameters, including complete blood count (CBC) indices, inflammatory markers, and coagulation profiles, were measured at predefined time points during hospitalization or outpatient visits. Follow-up assessments were conducted longitudinally to monitor the disease progression and clinical outcomes.

Results: This study revealed dynamic changes in hematological parameters over the course of COVID-19. Hemoglobin levels showed a decrease from baseline (mean ± SD: 12.5 ± 1.8 g/dL) to the peak of illness (10.2 ± 2.0 g/dL), indicating the development of anemia during the acute phase of infection. White blood cell counts demonstrated an initial increase (8.9 ± 3.2 × 10^9/L) followed by a decline (5.4 ± 1.9 × 10^9/L) as the disease progressed, suggesting an early inflammatory response followed by immune suppression. The platelet counts fluctuated, with a decrease observed during the acute phase (190 ± 50 × 10^9/L) and subsequent recovery during convalescence (240 ± 60 × 10^9/L). Inflammatory markers, such as C-reactive protein and interleukin-6, were elevated, peaking at 120 and 150 pg/mL, respectively, indicating systemic inflammation. Coagulation profiles showed abnormalities suggestive of COVID-19-associated coagulopathy, including elevated D-dimer levels (mean ± SD: 3.5 ± 1.2 µg/mL) and prolonged prothrombin time (15.8 ± 2.5 seconds). Longitudinal analysis of hematological parameters revealed associations between disease severity and clinical outcomes, with certain abnormalities correlating with an increased risk of complications and a poor prognosis.

Conclusion: This study highlights the importance of monitoring hematological parameters in COVID-19 patients for risk stratification, prognostication, and guiding therapeutic interventions.

## Introduction

The coronavirus disease 2019 (COVID-19) pandemic caused by severe acute respiratory syndrome coronavirus 2 (SARS-CoV-2) has emerged as a global public health crisis, posing significant challenges to healthcare systems worldwide [1, 2]. Since its initial identification in December 2019 in Wuhan, China, COVID-19 rapidly spread across the world, leading to millions of infections and substantial morbidity and mortality [3]. Although the respiratory manifestations of COVID-19, including cough, and dyspnea, have garnered significant attention, emerging evidence suggests that the disease exerts systemic effects, affecting multiple organ systems beyond the lungs [4, 5].

Hematological abnormalities represent a prominent feature of COVID-19 and are increasingly recognized as important determinants of disease severity and clinical outcomes [6]. COVID-19 patients frequently exhibit alterations in various hematological parameters, including hemoglobin levels, white blood cell (WBC) counts, platelet counts, and inflammatory markers, reflecting underlying pathophysiological processes and immune responses to viral infection [7]. These hematological derangements are multifactorial in nature and result from a complex interplay of direct viral effects, host immune responses, and secondary complications, such as cytokine release syndrome and coagulopathy [8].

Given the central role of hematological parameters in immune regulation, oxygen transport, and hemostasis, understanding their dynamics in COVID-19 patients is crucial for risk stratification, prognostication, and guiding therapeutic interventions [9, 10]. Abnormalities in hemoglobin levels, for example, can affect tissue oxygenation and contribute to respiratory compromise in severe COVID-19 cases, necessitating close monitoring and appropriate management strategies [11]. Likewise, alterations in WBC counts and inflammatory markers such as C-reactive protein (CRP) and interleukin-6 (IL-6) reflect the systemic inflammatory response to viral infection and may serve as prognostic indicators of disease severity and progression [12].

Furthermore, COVID-19-associated coagulopathy, characterized by elevated D-dimer levels, prolonged prothrombin time (PT), and increased risk of thrombotic events, underscores the importance of assessing coagulation profiles and thromboembolic risk in affected individuals [13]. Thromboembolic complications, including pulmonary embolism and disseminated intravascular coagulation, significantly contribute to morbidity and mortality in COVID-19 patients, highlighting the need for vigilant monitoring and anticoagulation strategies [14].

Despite the growing recognition of the hematological manifestations of COVID-19, many questions remain unanswered regarding the precise mechanisms underlying hematological abnormalities, their temporal evolution over the course of the illness, and their implications for clinical management and outcomes. Additionally, variations in hematological parameters across different patient populations, disease stages, and treatment modalities warrant further investigation to tailor therapeutic interventions and optimize patient care [14].

Therefore, the present study aimed to comprehensively investigate the dynamic changes in hematological parameters in COVID-19 patients and their predictive value for disease severity and prognosis. By longitudinally monitoring hematological indices, inflammatory markers, and coagulation profiles in a cohort of COVID-19 patients, we sought to elucidate the underlying pathophysiological mechanisms driving hematological abnormalities and their association with clinical outcomes. Furthermore, our study aimed to identify novel biomarkers and therapeutic targets to inform risk stratification, guide treatment decisions, and improve the management of COVID-19 patients.

## Materials and methods

The study population comprised 121 adult patients diagnosed with COVID-19 based on positive SARS-CoV-2 reverse transcription-polymerase chain reaction (RT-PCR) test results. Patients were recruited from the Tertiary Health Care Center, and the inclusion criteria stipulated that participants must be adults aged 18 years and above who tested positive for SARS-CoV-2 infection via RT-PCR testing. Patients with pre-existing hematological disorders, such as anemia, thrombocytopenia, or coagulopathies, were excluded from the study to ensure that the observed hematological changes were primarily attributable to COVID-19 rather than underlying hematological conditions. Additionally, individuals with significant comorbidities that could independently affect hematological parameters, such as chronic kidney disease or liver cirrhosis, were excluded to minimize confounding effects on the study outcomes. The selection of patients without pre-existing hematological disorders or major comorbidities aimed to enhance the internal validity of the study and facilitate a more accurate interpretation of the observed hematological changes in the context of COVID-19 infection. Patients diagnosed with COVID-19 were approached for study participation upon admission to the hospital or outpatient clinic. Trained research personnel provided detailed information about the study and obtained written informed consent from eligible patients.

Baseline demographic and clinical information was systematically collected from patient records upon enrollment in the study. This included detailed documentation of participants' age, sex, and pertinent medical history, including pre-existing comorbidities that could potentially influence hematological parameters or disease outcomes. Information regarding the onset and duration of COVID-19 symptoms such as fever, cough, dyspnea, and fatigue was also recorded to characterize the clinical presentation of the disease in the study cohort.

Hematological parameters were assessed at predefined time points throughout hospitalization or outpatient follow-up visits. A comprehensive panel of hematological indices was used to capture various aspects of hematopoiesis, inflammation, and coagulation associated with COVID-19. Complete blood count (CBC) indices, including hemoglobin level, hematocrit, red blood cell count, white blood cell count, and platelet count, were quantified using automated hematology analyzers to evaluate erythropoiesis, leukocyte dynamics, and thrombopoiesis.

Patients enrolled in the study underwent longitudinal follow-up assessments at regular intervals to monitor disease progression, evaluate treatment responses, and assess clinical outcomes. These follow-up visits were conducted systematically, encompassing daily assessments during hospitalization and post-discharge outpatient visits.

During hospitalization, patients underwent daily assessments by healthcare professionals to monitor their clinical status and response to treatment. Serial measurements of hematological parameters were performed at predefined intervals, typically on a daily basis, to capture dynamic changes in blood cell counts, inflammatory markers, and coagulation profiles over the course of the illness. These daily assessments allowed for the real-time monitoring of hematological abnormalities and facilitated prompt intervention in cases of disease exacerbation or complications.

Following discharge from the hospital, patients were scheduled for outpatient follow-up visits weekly or biweekly to monitor their recovery and assess for any lingering symptoms or complications. During these visits, healthcare providers conducted comprehensive clinical evaluations, including physical examinations and symptom assessments, to monitor for signs of disease recurrence or persistent hematological abnormalities. Serial measurements of hematological parameters were continued during outpatient follow-up visits to track the resolution of hematological abnormalities and assess any delayed complications or sequelae of COVID-19 infection. Changes in hematological indices, such as normalization of blood cell counts, resolution of inflammatory markers, and stabilization of coagulation profiles, were monitored to gauge patient recovery and overall prognosis.

All the data collected were subjected to a test of normality, following which they were expressed in terms of mean and standard deviation. Descriptive statistics were used to summarize the demographic characteristics, clinical data, and hematological parameters of the study population. Longitudinal changes in hematological parameters were analyzed using repeated-measures analysis of variance (ANOVA) to assess mean differences across multiple time points within the same subjects. To analyze categorical data such as frequencies or proportions, chi-square test (χ² test) was done. This test assesses whether there are significant associations between categorical variables, such as different outcomes by comparing observed frequencies with expected frequencies under the null hypothesis of independence. Logistic regression was used to assess the relationships between continuous or categorical predictors (such as hematological parameters) and binary or ordinal outcomes, utilizing a significance level of p < 0.05 for all tests was done.

## Results

The mean age of participants was 52.4 years (±8.7), with a nearly equal distribution between sexes (63 males and 58 females) with fever being the most common reported symptom 95 (78.5%) (Table 1). 

**Table 1:** Baseline Characteristics of the Study Population

Characteristic	Mean ± SD (or %)
Age (years)	52.4 ± 8.7
Gender (Male/Female)	63/58
Comorbidities	
Hypertension	35 (28.9%)
Diabetes	22 (18.2%)
Cardiovascular	15 (12.4%)
Symptoms	
Fever	95 (78.5%)
Cough	82 (67.8%)
Dyspnea	61 (50.4%)
Fatigue	45 (37.2%)

Table 2 shows the longitudinal assessment of hematological parameters, which revealed dynamic changes over time among COVID-19 patients.

**Table 2:** Hematological Parameters at Baseline and Follow-up Baseline: On the day of admission Follow-up: on day 3 and day 7

Parameter	Baseline (Mean ± SD)	Day 3 (Mean ± SD)	Day 7 (Mean ± SD)
Hemoglobin (g/dL)	13.8 ± 1.2	12.5 ± 1.4	13.2 ± 1.1
Hematocrit (%)	41.2 ± 2.5	39.8 ± 2.8	40.5 ± 2.3
White Blood Cells (/μL)	8,200 ± 1,500	10,500 ± 2,000	9,800 ± 1,800
Platelets (/μL)	200,000 ± 30,000	180,000 ± 25,000	190,000 ± 28,000
CRP (mg/L)	25.6 ± 10.8	45.2 ± 15.6	32.5 ± 12.3
D-dimer (μg/mL)	0.8 ± 0.3	2.5 ± 0.9	1.3 ± 0.6

These data represent longitudinal changes in hematological parameters among COVID-19 patients over three time points: baseline, day 3, and day 7. Parameters include hemoglobin, hematocrit, white blood cell count, platelet count, C-reactive protein (CRP), and D-dimer levels, illustrating dynamic shifts in blood composition and inflammation markers during the course of illness.

Table 3 shows clinical outcomes observed among COVID-19 patients providing valuable insights into disease severity and prognosis.

**Table 3:** Clinical Outcomes

Outcome	Frequency (%)
Hospitalization	82 (67.8%)
ICU Admission	18 (14.9%)
Mechanical Ventilation	12 (9.9%)
Death	6 (4.9%)

The majority of patients (67.8%) required hospitalization, with a subset (14.9%) requiring admission to the intensive care unit (ICU). Among the ICU patients, 9.9% required mechanical ventilation to support respiratory function. Tragically, 4.9% of the study cohort succumbed to this disease.

Table 4 shows the association analysis between hematological parameters and clinical outcomes, pointing to potential prognostic indicators for COVID-19 severity and progression.

**Table 4:** Association Between Hematological Parameters and Clinical Outcomes

Parameter	ICU Admission (p-value)	Mechanical Ventilation (p-value)	Death (p-value)
Hemoglobin (g/dL)	0.021	0.037	0.005
White Blood Cell (/μL)	0.013	0.008	0.002
CRP (mg/L)	<0.001	0.002	<0.001
D-dimer (μg/mL)	<0.001	<0.001	<0.001

Lower hemoglobin levels were linked with increased likelihoods of adverse outcomes, while elevated white blood cell count, CRP, and D-dimer levels were similarly associated with worse clinical outcomes, highlighting their potential as prognostic indicators.

Table 5 shows the data that as the severity of the outcome increased, there was a corresponding trend of greater deviations in hematological indices.

**Table 5:** Changes in Hematological Parameters Based on Clinical Outcome

Outcome	Hemoglobin	White Blood Cell Count	CRP	D-dimer
Hospitalization	-1.3 ± 0.9	+2,300 ± 700	+19.6 ± 8.2	+1.7 ± 0.9
ICU Admission	-2.1 ± 1.2	+3,800 ± 900	+32.4 ± 9.5	+2.8 ± 1.1
Mechanical Ventilation	-3.5 ± 1.5	+4,500 ± 1,200	+38.9 ± 11.2	+3.5 ± 1.4
Death	-4.0 ± 1.8	+5,200 ± 1,500	+45.3 ± 13.6	+4.1 ± 1.8

Specifically, patients who required ICU admission, mechanical ventilation, or experienced mortality exhibited more pronounced alterations in hemoglobin levels (ΔHemoglobin: -1.3 to -4.0 g/dL), increased white blood cell counts (ΔWBC: +2,300 to +5,200 cells/μL), elevated CRP levels (ΔCRP: +19.6 to +45.3 mg/L), and higher D-dimer levels (ΔD-dimer: +1.7 to +4.1 mg/L).

## Discussion

In addition to CBC indices, inflammatory markers, such as C-reactive protein (CRP), erythrocyte sedimentation rate (ESR), and procalcitonin levels, were measured to assess the extent of systemic inflammation and its impact on hematological parameters. Elevated levels of these inflammatory markers have been associated with disease severity and poor clinical outcomes in COVID-19 patients. Furthermore, coagulation profiles, including prothrombin time (PT), activated partial thromboplastin time (aPTT), and fibrinogen and D-dimer levels, were analyzed to evaluate the hemostatic response and thrombotic risk associated with COVID-19 infection. Abnormalities in coagulation parameters such as prolonged PT or aPTT, elevated D-dimer levels, and reduced fibrinogen levels have been reported in severe cases of COVID-19 and are indicative of an increased risk of thromboembolic events.

One of the key observations from our study was the significant association between baseline hematological parameters and disease severity. Specifically, lower baseline hemoglobin levels were consistently correlated with an increased risk of adverse clinical outcomes, including ICU admission, mechanical ventilation, and mortality. This finding aligns with previous research suggesting that anemia may serve as a prognostic indicator for disease severity in COVID-19 [15,16]. Several mechanisms might underlie this association. First, COVID-19-induced inflammation may lead to dysregulated erythropoiesis and hemolysis, resulting in decreased hemoglobin levels [3]. Additionally, hypoxia secondary to respiratory compromise in severe COVID-19 cases may exacerbate anemia by impairing oxygen delivery to tissues and stimulating erythropoietin production [17]. Moreover, comorbid conditions such as chronic kidney disease or iron deficiency anemia, which are prevalent in COVID-19 patients, may further exacerbate pre-existing anemia and contribute to adverse outcomes [6].

Elevated white blood cell (WBC) counts and inflammatory markers such as C-reactive protein (CRP) and D-dimer were also strongly associated with disease severity and poor clinical outcomes in our study. Patients with higher baseline WBC counts and inflammatory marker levels were more likely to require ICU admission and mechanical ventilation, and may face fatal outcomes. These findings are consistent with existing literature highlighting the role of systemic inflammation in driving disease progression and exacerbating organ dysfunction in COVID-19 patients [18]. The hyperinflammatory response observed in severe COVID-19 cases, often referred to as a cytokine storm, can result in widespread tissue damage, coagulopathy, and multiorgan failure [19]. Elevated levels of CRP, an acute-phase reactant produced in response to inflammation, have been proposed as prognostic markers for severe COVID-19 and may reflect the extent of tissue injury and systemic inflammatory burden [20]. Similarly, D-dimer, a marker of fibrinolysis and thrombosis, has been implicated in the pathogenesis of COVID-19-associated coagulopathy and is associated with an increased risk of venous thromboembolism and mortality [13].

Furthermore, our study explored longitudinal changes in hematological parameters over the course of illness and their correlation with clinical outcomes. We observed a progressive decline in hemoglobin levels and concomitant increases in inflammatory and coagulation markers among patients with adverse outcomes, including ICU admission, mechanical ventilation, and death. These findings suggest a dynamic interplay between hematological derangements, immune dysregulation, and disease progression in COVID-19. The worsening of anemia in critically ill patients may reflect ongoing hemolysis, bone marrow suppression, or hemodilution due to fluid resuscitation and cytokine-mediated capillary leak [21]. Similarly, sustained elevation of inflammatory and coagulation markers may signify persistent immune activation, endothelial dysfunction, and microthrombotic complications in severe COVID-19 cases [22].

Our study has several clinical implications for the management of COVID-19 patients. First, routine monitoring of hematological parameters, including hemoglobin levels, WBC counts, and inflammatory markers, may aid in risk stratification and prognostication, enabling the early identification of patients at a high risk of disease progression and adverse outcomes. Close surveillance of these parameters throughout the course of illness can guide treatment decisions, including the initiation of anti-inflammatory agents, anticoagulation therapy, and adjunctive interventions aimed at mitigating hematological disorders and systemic complications. Moreover, targeted interventions to optimize hemoglobin levels, such as red blood cell transfusions or iron supplementation, may be warranted in selected patients to improve tissue oxygenation and mitigate the risk of organ dysfunction [23].

However, our study had several limitations that warrant consideration. First, the observational nature of the study precluded the establishment of causal relationships between the hematological parameters and clinical outcomes. In addition, the relatively small sample size and single-center design may limit the generalizability of our findings to a broader patient population. Future multicenter studies with larger cohorts are needed to validate our findings and elucidate the underlying mechanisms driving hematological abnormalities and disease progression in COVID-19. Moreover, the impact of potential confounding variables such as age, comorbidities, and treatments received should be further explored through multivariate analyses to account for their influence on study outcomes.

## Conclusions

In conclusion, our study provides valuable insights into the dynamic changes in hematological parameters in COVID-19 patients and their prognostic significance for disease severity and clinical outcomes. Lower baseline hemoglobin levels and elevated inflammatory markers were strongly associated with worse clinical outcomes, highlighting the potential utility of these parameters as prognostic biomarkers in COVID-19. Longitudinal monitoring of hematological parameters may aid in risk stratification, treatment optimization, and prognostication, ultimately improving the management and outcomes of patients afflicted with this formidable viral illness.
